# The Infection Route of Tomato Zonate Spot Virus in the Digestive System of Its Insect Vector *Frankliniella occidentalis*

**DOI:** 10.3389/fmicb.2022.911751

**Published:** 2022-06-28

**Authors:** Yong Chen, Yuyan Liu, Liang Wang, Heng Li, Tingting Linghu, Yixin Chen, Houjun Tian, Shuo Lin, Xue Zheng, Hui Wei

**Affiliations:** ^1^Fujian Key Laboratory for Monitoring and Integrated Management of Crop Pests, Fuzhou Scientific Observing and Experimental Station of Crop Pests of Ministry of Agriculture, Fujian Engineering Research Center for Green Pest Management, Institute of Plant Protection, Fujian Academy of Agricultural Sciences, Fuzhou, China; ^2^State Key Laboratory of Ecological Pest Control for Fujian and Taiwan Crops, Fujian Agriculture and Forestry University, Fuzhou, China; ^3^Institute of Biotechnology and Germplasm Resources, Yunnan Academy of Agricultural Sciences, Kunming, China

**Keywords:** tomato zonate spot virus, thrips, digestive system, transmission mode, immunofluorescence, transmission electron microscopy

## Abstract

Tomato zonate spot virus (TZSV) is a phytopathogen of the genus *Orthotospovirus* (*Bunyaviridae*) that is widespread in many areas of Southwest China. TZSV is mainly transmitted by *Frankliniella occidentalis*, but its exact infection route remains unclear. To explore this issue, we detected the nucleocapsid protein of TZSV in the digestive systems of first-instar *F. occidentalis* nymphs fed with TZSV-infected pepper leaves. TZSV infection in the *F. occidentalis* digestive system begins within 4 h post-first access to diseased plants: The foregut is likely the primary site of infection, and primary salivary glands (PSGs) are the destination. There are three potential routes for TZSV transmission from the alimentary canal to the PSGs: (1) virus dissemination from the midgut to hemocoel followed by movement to the PSGs; (2) accumulation in midgut epithelial cells and arrival at PSGs *via* tubular salivary glands and efferent ducts; and (3) arrival at epitheliomuscular cells of the forepart of the midgut and movement along the ligament to the PSGs. We tested the transmission efficiency of *F. occidentalis* in second-instar nymphs and female and male adults. TZSV was transmitted in a persistent-propagative mode by both nymphs and adults, with adults appearing to show slightly higher transmission efficiency than nymphs. We confirmed the presence of all three routes for TZSV transmission in *F. occidentalis* and determined that like other *Orthotospoviruses*, TZSV is transmitted in a persistent-propagative manner. These results should facilitate the control of TZSV-related diseases and further our understanding of the transmission biology of *Orthotospoviruses* in general.

## Introduction

Tomato zonate spot virus (TZSV) is a recently identified species in the genus *Orthotospovirus* of the *Bunyaviridae* family belonging to the watermelon silver mottle virus (WSMoV) serogroup. TZSV was first isolated from infected tomato samples in Yunnan Province (Dong et al., 2008) and is now widespread in many regions of Southwest China. To date, 25 plant species from 7 families have been reported as hosts of TZSV, including such economically important crops as tomato (*Solanum lycopersicum*), potato (*Solanum tuberosum*), and pepper (*Capsicum annuum*) and several ornamental plants, including crinum (*Crinum asiaticum*) and iris (*Iris tectorum*; Dong et al., 2008, 2010; Li et al., 2014; Zheng et al., 2014; Huang et al., 2015; Liu et al., 2015; Wu et al., 2015). Thus, TZSV poses a threat to the local agricultural economy in Southwest China. Plant infection with TZSV is often associated with consistent symptoms, such as concentric zoned rings pots and necrotic lesions on fruits, leaves, and stems (Dong et al., 2008; Zhao and Wang, 2012).

*Orthotospoviruses* rely on different species of thrips for horizontal transmission among plant hosts. These viruses are ingested into the digestive canals of thrips vectors and enter the primary salivary glands (PSGs) before being transmitted to plant hosts (Rotenberg et al., 2015). According to studies of the tomato spotted wilt virus (TSWV)–*Frankliniella occidentalis* model system, TSWV must overcome several barriers, such as the midgut infection barrier (Nagata et al., 1999) and midgut escape barrier (Ullman, 1992) before it is successfully transmitted. The infection of PSGs always occurs after the infection of alimentary canals (Kritzman et al., 2002), and viral particles have been observed in the hemocoel of viruliferous *F. occidentalis* (Ullman, 1992). The ligaments connect the anterior midgut with the PSGs. The tubular salivary glands (TSGs) connecting the middle part of the midgut with the PSGs also enable viral entry into the PSGs (Nagata et al., 1999; de Assis Filho et al., 2002; Montero-Astúa et al., 2016).

TZSV can be transmitted by four species of thrips: *F. occidentalis*, *F. schultzei*, *Thrips palmi* and *T. tabaci* (Dong et al., 2008; Zhao and Wang, 2012; Zheng et al., 2014; Chen et al., 2021). *F. occidentalis*, the dominant thrips species in several areas of China, is also the main vector of TZSV (Zheng et al., 2014; Yin et al., 2018; He et al., 2020). Much is known about the behaviors and related infection mechanisms of TZSV in thrips vectors (Zheng et al., 2014, 2019; Chen et al., 2021), whereas the infection route of TZSV in thrips vectors remains poorly understood.

Based on the transmission of other *Orthotospoviruses*, we propose three potential routes of TZSV infection of PSGs: (1) the virus passes through the outer membrane of the epitheliomuscular cells and remains in the hemolymph until its arrival at the PSGs (Ullman, 1992); (2) the virus reaches the anterior region of the epitheliomuscular cells of midgut 1 (Mg1) and then moves to the salivary cells of the PSGs *via* the ligaments that connect Mg1 to the PSGs (Nagata et al., 1999; Montero-Astúa et al., 2016); and (3) the TSGs connecting Mg2 to the PSGs may play an intermediary role in viral delivery (Montero-Astúa et al., 2016).

In this study, we used confocal laser scanning microscopy (CLSM) and transmission electron microscopy (TEM) to study the infection route of TZSV in its insect vector *F. occidentalis*. The results increase our understanding of the transmission mechanism of *Orthotospoviruses*, provide a fundamental basis for further studies of the interaction between TZSV and its thrips vectors, and may facilitate the control of the associated diseases.

## Materials and Methods

### Virus, Plant, Antibodies, and Insects

Samples of tomato (*S. lycopersicum*) infected with TZSV were provided by the Institute of Biotechnology and Germplasm Resources, Yunnan Academy of Agricultural Sciences, Kunming 650,205, China. The virus was mechanically inoculated onto pepper (*C. annuum*, Zunla-1) leaves. Healthy pepper plants were obtained locally and grown in a greenhouse maintained at 25°C under natural light conditions and used for virus transmission by thrips. A polyclonal antibody against the nucleocapsid protein of TZSV (TZSV-N) was prepared in rabbit as previously described (Jia et al., 2012a). The actin dye phalloidin-rhodamine was purchased from Invitrogen. IgGs isolated from the polyclonal antibodies were conjugated to fluorescein isothiocyanate (FITC; Invitrogen) according to the manufacturer’s instructions.

*F. occidentalis* thrips were originally donated by the Institute of Plant Protection, Hunan Academy of Agricultural Sciences, China. The insects were reared on broad beans (*Vicia faba*) in a climate chamber (25°C; 65 ± 5% relative humidity and a light: dark regime of 14 h: 10 h) as described previously (Lin et al., 2021). Adult thrips used for reproduction were collected every 24 h in a clean container to allow them to spawn on freshly soaked beans.

### Dissection of the Digestive System

A drop of phosphate-buffered saline (PBS) solution (0.01 M, pH7.4) was placed on the surface of a slide. Thrips were chilled on ice for 20 s and transferred to the drop of PBS, and the digestive system was dissected under an optical microscope. A dissecting needle was pressed onto the hind part of each thrips to hold it tightly, another dissecting needle was used to carefully remove the head, and the digestive system was extruded through the abdomen.

### Immunofluorescence Assays

To visualize the viral infection cycle in *F. occidentalis*, first-instar *F. occidentalis* nymphs (12 h old) were fed for an acquisition access period of 12 h on TZSV-infected pepper leaves and transferred to freshly soaked beans. At different times (1, 4, 24, 48, 72, 144, or 216 h) post-first access to diseased plants (padp), 40 thrips digestive organs were dissected, fixed, and immunolabeled before observation. *F. occidentalis* fed with healthy pepper leaves was used as a negative control. The digestive organs were dissected carefully in PBS solution (0.01 M, pH7.4) under an optical microscope. The PBS was removed, and the samples were fixed with 4% paraformaldehyde (PFA) for 2 h and subsequently permeabilized with 16% PFA (Alfa Aesar, United States) for 1 h at room temperature. After removing of the Triton solution and subsequent washing steps, the tissues were incubated with anti-rabbit antibodies against the nucleocapsid protein of TZSV (TZSV-N) conjugated to fluorescein isothiocyanate (FITC) and the actin dye phalloidin-rhodamine (Invitrogen) in PBS supplemented with 3% bovine serum albumin (BSA) for 2 h at 37°C. The samples were washed three times with PBS and placed on a clean slide. Anti-fade mounting medium (20 μl) with DAPI (Vectashield) was added to the guts and salivary glands, which were then covered with a coverslip. The slides were kept at 4°C and protected from light before being processed for immunofluorescence microscopy (Leica SP8, Germany).

### Hemolymph Collection

To examine the ability of TZSV to overcome the membrane barriers of the midgut to complete its infection route, the following method was used to extract the hemolymph from TZSV-infected *F. occidentalis* (Liu et al., 2006; Jia et al., 2012b). First-instar thrips were fed on TZSV-infected pepper leaves for 12 h for viral acquisition and transferred to freshly soaked beans. At 48 h padp, 30 thrips were selected and chilled on ice for 2 min. A drop of PBS solution was placed onto the surface of a Polysine slide (Epredia, United States). All six legs were removed from each thrips, and the wounded body was soaked rapidly in PBS, which helped the hemolymph flow from the wounds into the PBS. The collected hemolymph was then fixed with 4% PFA for 20 min, permeabilized with 2% Triton X-100 for 30 min, incubated with anti-TZSV-N antibodies and DAPI, and observed under the CLSM to monitor the presence of TZSV-N. In addition, some of the isolated hemolymph samples were used for RNA extraction and PCR analysis to confirm the presence of TZSV.

### TEM Analysis

The guts and salivary glands of TZSV-infected *F. occidentalis* were dissected and fixed with 1% glutaraldehyde and 4% PFA in PBS overnight at 4°C. The fixed samples were washed three times with PBS on ice and dehydrated through an ethanol gradient (30, 50, 50, 70, 90, 95, 100, and 100%) at −20°C. The dehydrated tissues were permeated with a graded series (30, 50, and 100%) of LR Gold Resin (Bioscience) at −20°C and embedded in 0.13% benzil in LR Gold Resin. All samples were polymerized under ultraviolet light at −20°C for 3 days. The samples were sectioned on an ultramicrotome (Leica UC5, Germany) with a diamond knife and incubated with rabbit antiserum against TZSV-N and immunogold-labeled anti-goat antibodies against rabbit IgG that had been conjugated with 6-nm gold particles (Jackson). The sections were stained with 2% uranyl acetate, followed by 3% lead citrate for 10 min each time and observed under an electron microscope (HitachiH-7,700, Japan).

Immunogold labeling negative staining with TZSV-N-specific rabbit polyclonal antibody was used to identify viral particles in the sap of TZSV-infected pepper leaves as a positive control as previously described (Zhang et al., 2016). The samples were observed under an electron microscope (HitachiH-7,700, Japan).

### Transmission Efficiency Assay

First-instar nymphs (12 h old) were fed on TZSV-infected pepper leaves for a 12-h viral acquisition period and then transferred to a transparent container, and reared on freshly soaked beans. The top of the container was covered with two layers of Parafilm. At approximately 48 h padp, 20 second-instar nymphs were chosen and placed individually into 50-mL tubes containing a healthy pepper plant (four leaf stage). The plants were inoculated for 48 h with potentially viruliferous thrips. In addition, at ~192 h padp, the rest of first-instar nymphs (which were given a 12-h TZSV acquisition period) had grown into adults. Subsequently, female and male potentially viruliferous thrips were collected individually to test the TZSV transmission efficiency as described previously. After the inoculation access period, the thrips were removed, and the inoculated test plants were maintained at 25°C under natural light conditions for 240 h prior to the RT-PCR assay. Total RNA was extracted from pepper plants or thrips as described previously (Chen et al., 2016), and the viruliferous rate and transmission rate were measured by RT-PCR with TZSV-N-encoding gene primers (F: 5′-AAAGATTCAAGAACTATTGGCT-3′; R: 5′-TCTCAGTGAACTCCACGCTA-3′). Transmission rate (%) = (number of TZSV-infected pepper plants/number of TZSV-infected thrips) × 100. The data were analyzed by one-way analysis of variance (ANOVA) using IBM SPSS statistics 20. The experiment was performed in triplicate.

## Results

### Morphology of the Digestive System of *Frankliniella occidentalis*

**Figure 1:**
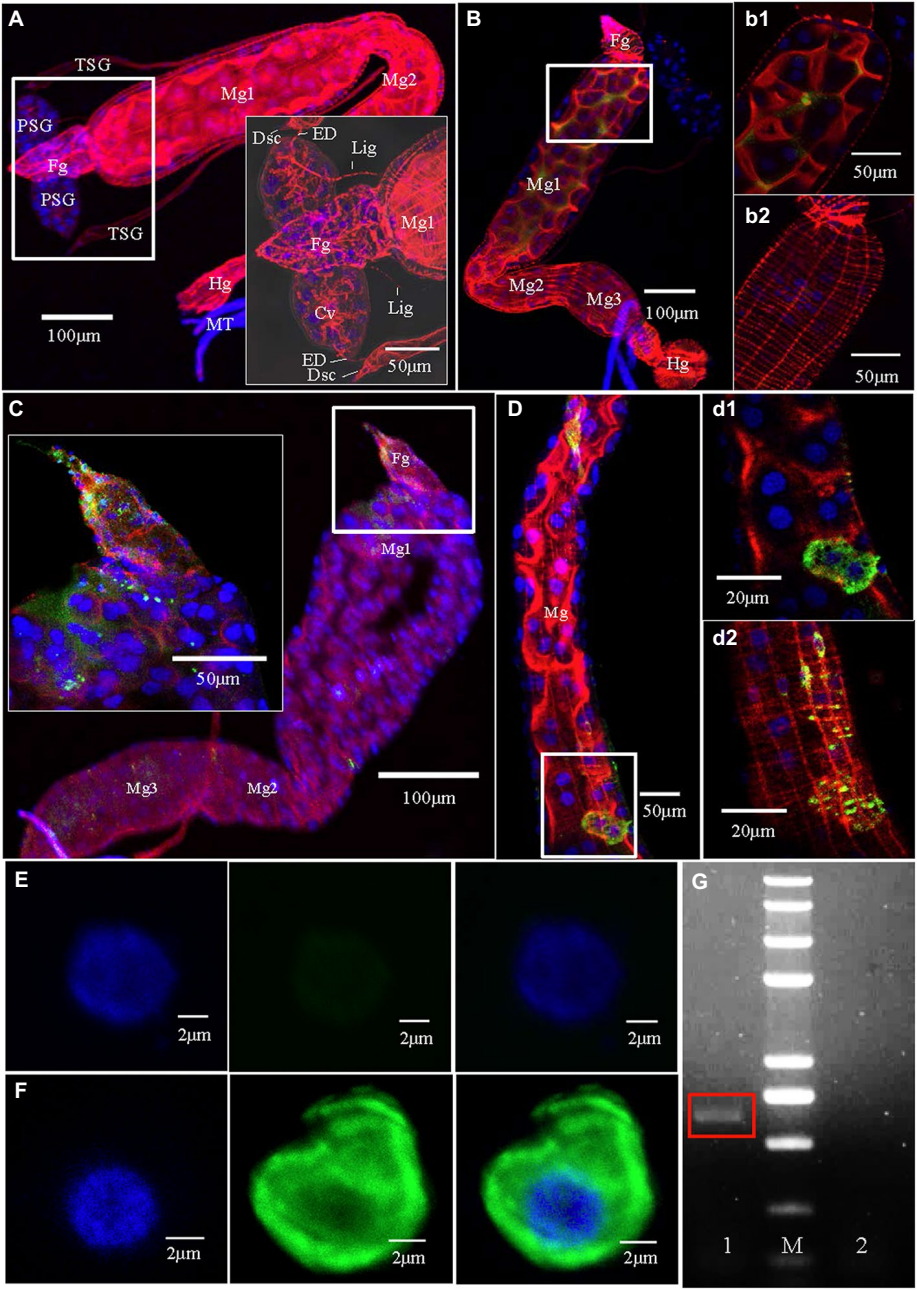
Tomato zonate spot virus (TZSV) infection routes in the body of *Frankliniella occidentalis*. Samples were immunolabeled with the actin dye phalloidin-rhodamine (red), the nuclear dye DAPI (blue), and the nucleocapsid protein of TZSV (TZSV-N) conjugated to fluorescein isothiocyanate (green) and observed under a confocal laser scanning microscope. **(A)** The digestive system of a second-instar nymph fed with healthy pepper leaves. The boxed area shows the morphological characteristics of the upper portion of the digestive system. **(B)** At 1 h post-first access to diseased plants (padp), TZSV initially entered the gut lumen; (b1) and (b2) are enlargements of the boxed areas in **(B)** and show the presence of TZSV in the midgut lumen (b1) but not in muscle tissues (b2). **(C)** At 4 h padp, a small amount of TZSV immediately entered the epithelial cells of the foregut and the anterior portion of the midgut; the enlarged boxed area shows TZSV location in greater detail. **(D)** At 24 h padp, intense TZSV-N fluorescent signal was observed in the epithelial cells (d1) and the epitheliomuscular cells (d2) of the midgut. **(E)** A series of images showing a hemocyte of *F. occidentalis* fed with healthy pepper leaves; scale bars, 2 μm. **(F)** Detection of the distribution of TZSV-N in the hemocyte of an infected *F. occidentalis* at 72 h padp. **(G)** Agarose gel electrophoresis of the RT-PCR product of the TZSV-N-encoding gene in *F. occidentalis* hemolymph. Lane 1, TZSV-infected *F. occidentalis*; M, 2-kb DNA ladder; lane 2, uninfected *F. occidentalis* control.

**Figure 2:**
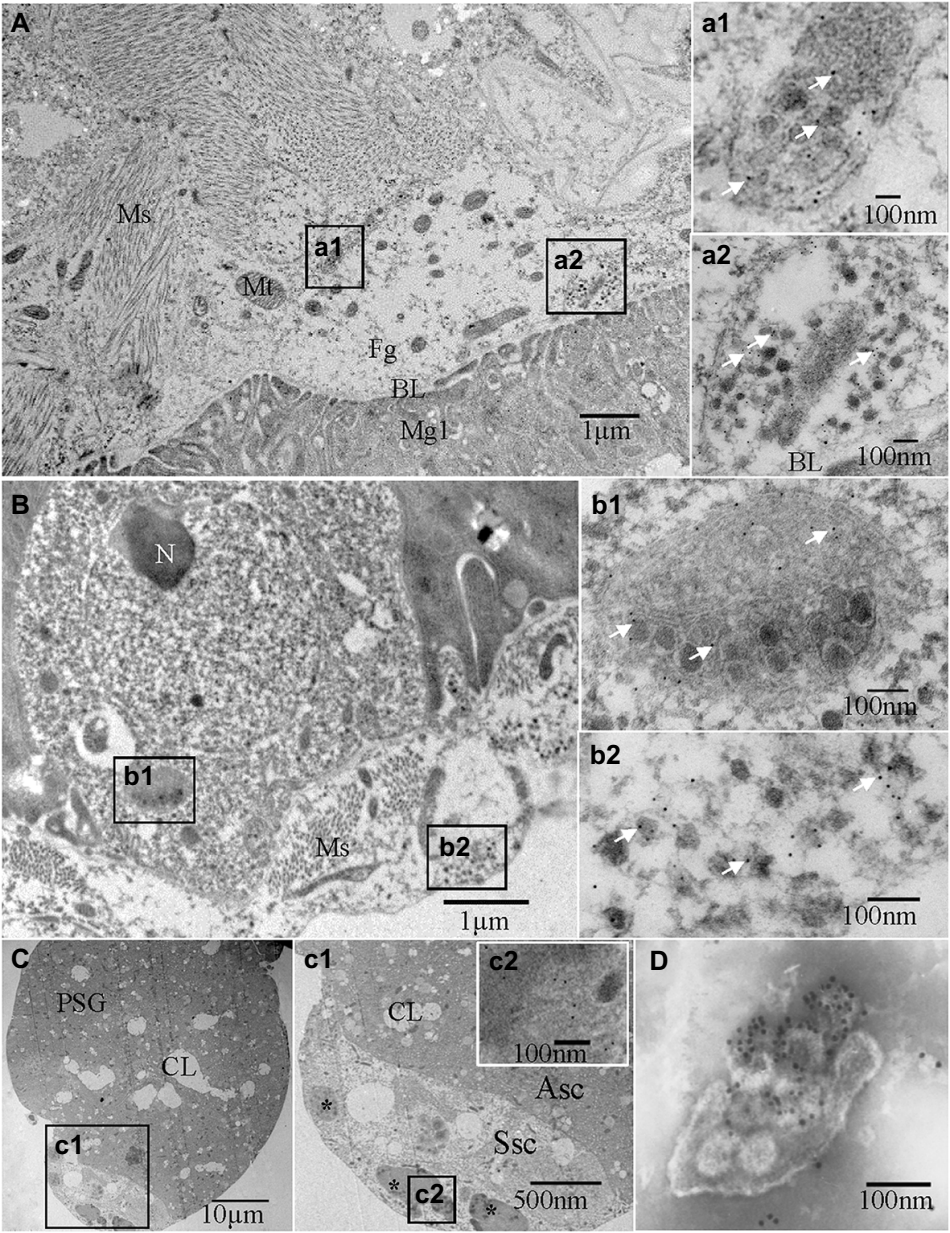
Immunoelectron micrographs showing the subcellular distribution of TZSV particles and the TZSV nucleocapsid protein N (TZSV-N) in digestive systems of 2nd instar *Frankliniella occidentalis* nymphs at 72 h padp. Tissues were immunolabeled with TZSV-N-specific IgG as the primary antibody, followed by a specific secondary antibody that had been conjugated with 6-nm gold particles (white arrows). **(A)** Localization of viral particles in the foregut. (a1) and (a2) are enlargements of the boxed area in **(A)**, showing viral clustering in vesicles in the foregut; (a2) vesicle membrane fusing within the basal lamina between the foregut and midgut. **(B)** Epithelial cells and associated epitheliomuscular cells of the midgut. (b1) and (b2) are enlargements of the boxed area in **(B)**, showing details of the morphological characteristics and localization of viral particles and TZSV-N. (C) PSGs of *F. occidentalis*. (c1) is an enlargement of the boxed area in **(C)**, showing the characteristics of a senescent secretory cell and the localization of TZSV viroplasma (^*^); (c2) is an enlargement of the boxed area in (c1), showing gold particles on the electron-dense viroplasm matrix in a senescent secretory cell of the a PSG. **(D)** TZSV virion obtained from the sap of infected pepper leaves; 12-nm gold particles specifically labeled the TZSV particles. Asc: active secretory cell; BL: basal lamina; CL: cistern lumen; Fg: foregut; Hg: hindgut; Mg: midgut; Ms.: muscle; Mv: microvilli; N: nucleus; PSG: primary salivary gland; Ssc: senescent secretory cell; TSG: tubular salivary glands.

**Figure 3:**
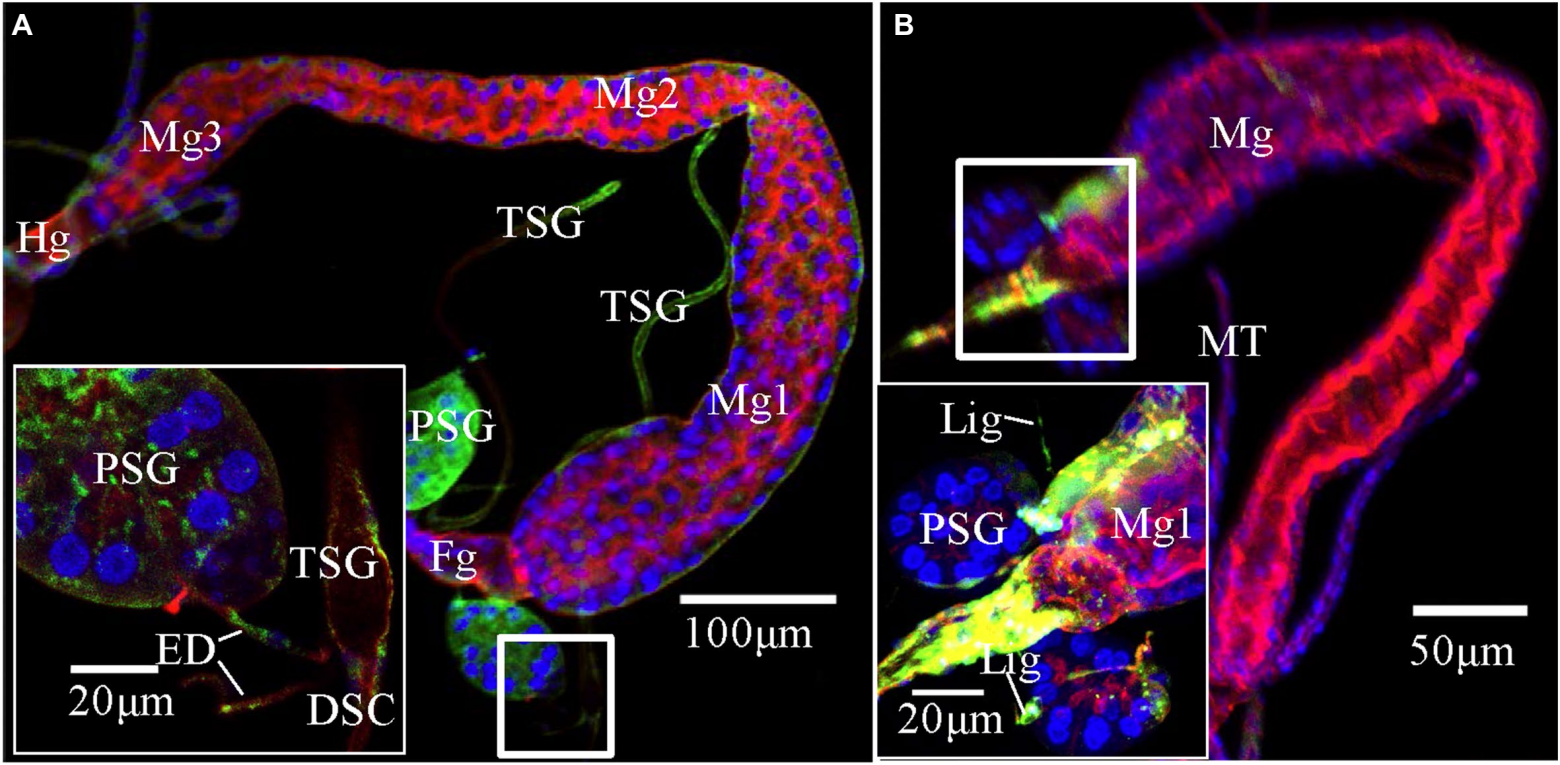
Potential route of tomato zonate spot virus (TZSV) infecting the primary salivary glands (PSGs) of *Frankliniella occidentalis*. TZSV-infected samples were immunolabeled with the actin dye phalloidin-rhodamine (red), the nuclear dye DAPI (blue), and the nucleocapsid protein of TZSV (TZSV-N) conjugated to fluorescein isothiocyanate (green) and observed under a confocal laser scanning microscope. **(A)** TZSV-N locates to the midgut 2, hindgut, PSGs, and efferent ducts connecting PSGs and tubular salivary glands (TSGs; second instar, 48 h padp). The boxed area indicates the localization of the virus in the efferent ducts, TSGs, and PSGs. **(B)** TZSV-N localizes to the foregut, midgut 1, PSGs, and ligaments connecting the PSGs and midgut 1 (second instar, 48 h padp). Abbreviations: DSC, deferent salivary canal; ED, efferent duct; Fg, foregut; Lig, ligament; Mg, midgut; MT, Malpighian tubule; PSG, primary salivary gland; TSG, tubular salivary gland.

To better understand the infection route of TZSV within its dominant insect vectors, we dissected the digestive organs of *F. occidentalis* infected (or not) with TZSV and examined them by CLSM and TEM (Figures 1–3). The digestive system of *F. occidentalis* contains a pair of oval PSGs; two long, slim TSGs; four Malpighian tubules (MTs); and a main digestive tract consisting of a conical foregut, tubular midgut, and short hindgut. The midgut is further divided into three parts: midgut 1 (Mg1; anterior), midgut 2 (Mg2; central), and midgut 3 (Mg3; posterior; Figures 1A,B). The PSGs are composed of several binuclear and mononuclear cells and contain a large central cistern lumen. The pair of PSGs is connected to the anterior portion of the midgut by a pair of tenuous ligaments. The efferent ducts located at the tips of TSGs are also linked with the apical regions of the PSGs (Figure 1A).

### The TZSV Infection Route in Digestive Organs

We investigated the dynamics of TZSV infection in the digestive system of *F. occidentalis* (Table 1). More than 500 first-instar nymphs of thrips were fed with virus-infected pepper leaves for 12 h and transferred to freshly soaked beans. At 1, 4, 24, 48, 72, 144, and 216 h after viral acquisition, 40 digestive organs were dissected from the thrips and processed for immunofluorescence microscopy. We observed TZSV fluorescent signals in virus-infected digestive organs, whereas we detected no FITC signals in the digestive organs of *F. occidentalis* fed with healthy pepper leaves (Figure 1A). At 1 h padp, the weak green signal of TZSV-N was present only in some of the midgut lumen, which could be considered a prelude to further infection (Figure 1B). Signals began to be detectable the epithelial cells at 4 h padp, and the infection rate increased from 0% at 1 h padp to 62.50% at 4 h padp. TZSV was detected in 60.00% of the dissected foreguts, and the signals appeared as several bright green spots localized to the epithelium (Figure 1C). Moreover, the infection rates of TSGs (42.50%), PSGs (37.50%), and Mg1 (30.00%) indicated that TZSV particles rapidly infected the PSGs (Table 1). Using immunogold labeling with TZSV-N-specific rabbit polyclonal antibody, we observed dense TZSV-N proteins localized to the surfaces of virion particles (Figure 2D). Electron micrographs of the foregut and anterior midgut of TZSV-infected *F. occidentalis* showed that viral particles clustered into small groups in vesicles that remained in the foregut (Figure 2a1). Furthermore, the membrane of one vesicle was fused with the junction between the foregut and midgut (Figure 2a2).

**Table 1:** Percentage of tomato zonate spot virus (TZSV)-infected digestive tissues of *Frankliniella occidentalis*.

Digestive tissue^*^	First-instar nymphs				Second-instar nymphs	Pre-pupae/pupae	Adults
	1 h	4 h	24 h	48 h	72 h	144 h	216 h
Any digestive tissue part (Total infection rate)	0.00 (0)	62.50 (25)	80.00 (32)	85.00 (34)	82.50 (33)	85.00 (34)	87.25 (35)
Foregut	0.00 (0)	60.00 (24)	80.00 (32)	82.50 (33)	80.00 (32)	85.00 (34)	82.50 (33)
Midgut 1	0.00 (0)	30.00 (12)	27.50 (11)	62.50 (25)	60.00 (24)	75.00 (30)	87.25 (35)
Midgut 2	0.00 (0)	22.50 (9)	15.00 (6)	75.00 (30)	75.00 (30)	77.50 (31)	85.00 (34)
Midgut 3	0.00 (0)	12.50 (5)	17.50 (7)	57.50 (23)	65.00 (26)	60.00 (24)	70.00 (28)
Hindgut	0.00 (0)	10.00 (4)	12.50 (5)	30.00 (12)	35.00 (14)	50.00 (20)	67.50 (27)
Malpighian tubules	0.00 (0)	25.00 (10)	10.00 (4)	45.00 (18)	62.50 (25)	60.00 (24)	75.00 (30)
Tubular salivary glands	0.00 (0)	42.50 (17)	65.00 (26)	62.50 (25)	70.00 (28)	65.00 (26)	67.25 (27)
Primary salivary glands	0.00 (0)	37.50 (15)	42.50 (17)	57.50 (23)	60.00 (24)	62.50 (25)	65.00 (26)

* First-instar *F. occidentalis* nymphs (12 h old) were fed for an acquisition access period of 12 h on TZSV-infected pepper leaves and transferred to freshly soaked beans. Forty digestive organs were dissected at 1, 4, 24, 48, 72, 144, and 216 h post-first access to diseased plants. TZSV-N-FITC was used to indicate the presence of TZSV by green fluorescence. The percentage of infection was determined for each tissue examined. Data in parentheses indicate the number of virus-positive samples.

To determine the TZSV infection rates in the digestive tissues, we detected the presence of TZSV-N-FITC fluorescence. Data from 24 h padp showed a high infection rate in the foregut (80.00%), TSGs (65.00%), and PSGs (42.50%) compared to the data from 4 h padp, and the total infection rate increased to 80.00%. More viral particles clustered in the epithelium and the adjoining epitheliomuscular cells (Figures 1D, 2B). However, the infection rates of Mg2, Mg3, the hindgut, and MTs were less than 30% at both 4 and 24 h padp. At 48 and 72 h padp, the TZSV-N signals were dispersed among different digestive tissues. The total infection rates at 48 and 72 h padp were 85.00 and 82.50%, respectively, and the infection rate of each part of the digestive system exceeded 30.00%. Electron micrographs of *F. occidentalis* at 72 h padp showed that TZSV-N labeled with 6-nm gold particles was located in the mass of viroplasm structures in the senescent secretory cells of the PSGs (Figure 2C), indicating that the PSGs were a site of viral replication. At 144 and 216 h padp, the total infection rate was maintained at approximately 85.00% (Table 1).

### Potential Route of TZSV Infection of PSGs

There are three potential routes of infection of thrips PSGs by *Orthotospoviruses* (Ullman, 1992; Nagata et al., 1999; Kritzman et al., 2002; de Assis Filho et al., 2002; Montero-Astúa et al., 2016). First, we determined the presence of TZSV in the hemocoel by performing immunofluorescence assays. TZSV-N (green signal) was detected in the hemolymph cells of TZSV-infected *F. occidentalis* (Figure 1F) after 72 h padp, but no signal was detected in the hemolymph of the TZSV-uninfected *F. occidentalis* controls (Figure 1E). RT-PCR also demonstrated TZSV infection in the hemolymph of TZSV-infected *F. occidentalis*, indicating that TZSV could pass through the midgut barrier into the hemocoel.

We reanalyzed all images from the immunofluorescence assay and counted the percentage of infection in the midgut, foregut, ligaments, and TSGs in all samples that contained infected PSGs. In total, we counted 130 TZSV-infected samples containing TZSV-positive PSGs (Table 2). In these samples, the TZSV-N signal was mainly localized to the foregut, midgut, and TSGs, and the percentage of virus infection was 84.62, 87.69, and 80.77%, respectively. Efferent ducts that connect TSGs and PSGs are important conduits of viral movement from the midgut to PSGs (Montero-Astúa et al., 2016). Of the efferent ducts examined, 73.85% contained TZSV. Green TZSV-N signals were simultaneously detected in the TSGs, PSGs, and efferent ducts (Figure 3A). The ligaments connecting Mg1 and PSGs were also found to be a secondary route for viral movement. By contrast, the percentage of TZSV in the ligaments was much lower: We observed green signal representing TZSV-N localization in only 12 of 130 pairs of ligaments. The related morphological characteristics are shown in Figure 3B. Therefore, transmission *via* ligaments might be a less important route of TZSV movement to PSGs (Table 2).

**Table 2:** Localization of tomato zonate spot virus (TZSV) in different parts of the digestive systems of *Frankliniella occidentalis* showing TZSV-infected primary salivary glands (PSGs; *n* = 130).

Structure	Infection rate^*^
Foregut	84.62 (110)
Midgut	87.69 (114)
Ligaments connecting midgut 1 and PSGs	9.23 (12)
Tubular salivary glands (TSGs)	80.77 (105)
Efferent ducts connecting TSGs and PSGs	73.85 (96)

* The percentage of infection in the midgut, foregut, ligaments, and TSGs of immunofluorescence assay images containing infected PSGs was determined. TZSV-N-FITC was used to indicate the presence of TZSV by green fluorescence. Numbers in parentheses indicate the number of TZSV-positive samples.

### Transmission Efficiency of TZSV by *Frankliniella occidentalis*

To test whether *F. occidentalis* transmits TZSV persistently, like other *Orthotospoviruses*, and to compare the transmission efficiency of different metamorphosis stages, we collected first-instar nymphs and fed them with TZSV-infected pepper leaves for 48 h for viral acquisition. We then transferred the nymphs to freshly soaked beans as a food source and reared them for 2 or 8 days. At these time points, we transferred the second-instar nymphs and adult females and males individually to 50-mL Eppendorf (EP) tubes containing a healthy pepper plant (four leaf stage) and performed a virus transmission assay (Table 3). At these two metamorphosis stages, *F. occidentalis* was able to transmit TZSV into healthy pepper plants in a persistent transmission mode. The transmission rates by female adults (46.67 ± 4.41) and male adults (51.67 ± 6.67) appeared to be slightly higher than that by nymphs (41.67 ± 3.33), but these differences were not statistically significant (*p* > 0.05).

**Table 3:** Transmission efficiency (mean percentage ± SE) of tomato zonate spot virus (TZSV) by *Frankliniella occidentalis* at different developmental stages.

Metamorphosis stage	Mean level^*^	Replicate 1	Replicate 2	Replicate 3
Second-instar nymphs	41.67 ± 3.33a	45.00 (9/20)	45.00 (9/20)	35.00 (7/20)
Female adults	46.67 ± 4.41a	55.00 (11/20)	40.00 (8/20)	45.00 (9/20)
Male adults	51.67 ± 6.67a	45.00 (9/20)	65.00 (13/20)	45.00 (9/20)

* The transmission efficiency was calculated as the percentage of thrips and plants that tested positive for TZSV in RT-PCR assays. Numbers in parentheses indicate the number of TZSV-positive samples detected as a proportion of the total number of samples analyzed. Data in column 1 represent the mean ± standard deviation (SD) of the results from three replicates. Means followed by different letters differ significantly at *p <* 0.05.

## Discussion

Like most plant viruses, *Orthotospoviruses* require specific insect vectors for their effective long-distance transmission (Whitfield et al., 2015). Thrips are the primary vectors for *Orthotospoviruses*, and they also serve as important hosts that provide sites for viral replication (Rotenberg et al., 2015; Gupta et al., 2018; Rotenberg and Whitfield, 2018). Understanding the interactions between thrips and *Orthotospoviruses* is a key to controlling the diseases caused by these viruses, which have been the focus of much research (Zhao and Rosa, 2020; Chen et al., 2021; Mou et al., 2021).

To better understand the transmission route of TZSV through the digestive system of its dominant insect vector *F. occidentalis*, we performed a series of transmission experiments. The transmission electron micrographs from immunolabeling experiments showed that TZSV particles mainly formed small clusters that were enclosed within vesicles in the digestive system. Many vesicles were observed adjacent to the basal lamina between the foregut and midgut epithelial cells (Figure 2A). The viral particles could move intercellularly in an exocytosis-like manner (Figure 2a2). Viral particles could first enter the epithelial cells of the foregut or midgut and then be transmitted very rapidly into the adjacent epithelial or epitheliomuscular cells (Table 1). This suggests that TZSV can propagate well and circulate in its vector *F. occidentalis*. TZSV particles also showed a similar cellular distribution pattern in the cytoplasm as in the plant host of *F. occidentalis*: They were usually present as single particles or small groups of particles that clustered within the cytoplasm or at the ER membrane (Zhang et al., 2016).

**Figure 4:**
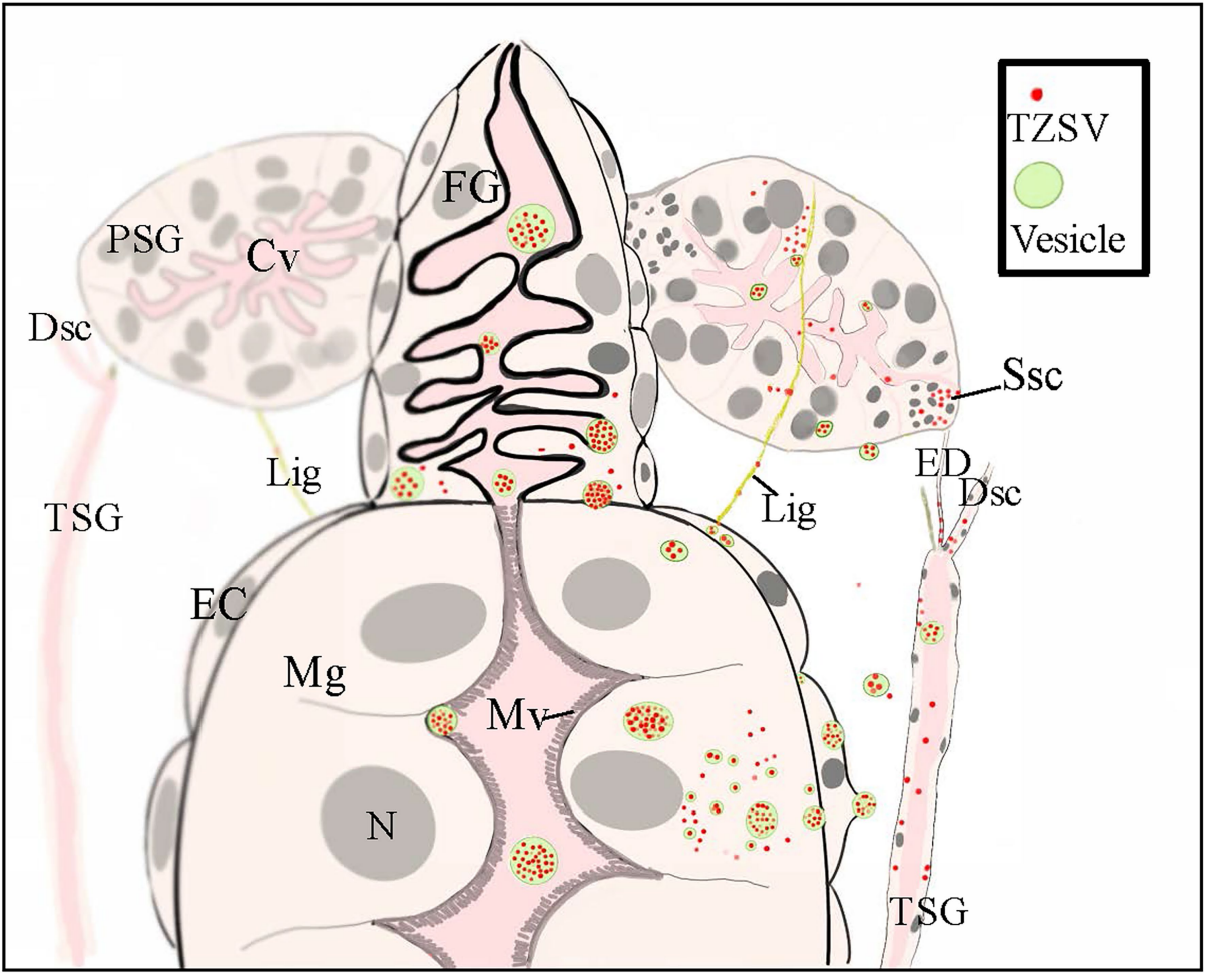
A schematic illustration showing the internal anatomy of the front part of the digestive system of nymphs of a *Frankliniella occidentalis* nymph and the connections between the primary salivary glands (PSGs) and other parts of the digestive system. The three postulated tomato zonate spot virus (TZSV) transmission routes are also depicted. (1) At the anterior-most region of the midgut, two slim ligaments connect each PSG and serve as a conduit for viral movement from the epitheliomuscular cells of the midgut to the secretory cells of PSGs. (2) TZSV particles can also enter epithelial cells of the foregut and midgut *via* endocytosis, replicate in the cells, or move directly into the epitheliomuscular cells and then the hemolymph before arriving at the secretory cells of PSGs. (3) The efferent duct connects the tubular salivary gland (TSG) with the corresponding PSG and serves as a conduit for viral movement from the TSG to the senescent secretory cells of PSGs. Abbreviations: CL, cistern lumen; DSC, deferent salivary canal; ED, efferent duct; Fg, foregut; Lig, ligament; Mg, midgut; EC: epitheliomuscular cell; Mv, microvillus; N, nucleus; Ssc, senescent secretory cell; PSG, primary salivary gland; TSG, tubular salivary gland.

The three routes for TZSV movement from the alimentary canal to the PSGs are depicted in Figure 4: (1) dissemination from the epitheliomuscular cells to the hemocoel, followed by movement to the PSGs; (2) accumulation in the epithelial cells of Mg1 and Mg2 and transfer into the apical regions of PSGs through the TSGs and efferent ducts; and (3) arrival at the epitheliomuscular cells of the forepart of Mg1, followed by movement along the “rope”-like ligaments to the secretory cells of the PSGs. Due to the difficulty in collecting hemolymph, we could not obtain data for the infection rate of hemolymph alone. However, transmission *via* the ligaments might be less important than the other two transmission mechanisms (Table 2). Given the higher infection rates of TSGs and efferent ducts, we hypothesize that the primary mechanism of TZSV transmission from the alimentary canal to the PSGs occurs *via* the TSGs. Previous studies suggested a fourth hypothetical mode of *Orthotospoviruses* movement from the midgut to the PSGs that depends on the ontogeny of the thrips (Moritz et al., 2004). Based on images of the positions of the internal organs of *F. occidentalis* at different developmental stages, the movement of *Orthotospoviruses* was proposed to occur during metamorphosis, when direct contact exists between the digestive organs. This might explain the observation that adult thrips can transmit some *Orthotospoviruses* only when they are infected at the nymph stage (Whitfield et al., 2005).

Four transmission modes have been categorized in virus–vector interactions: non-persistent, semi-persistent, persistent-circulative, and persistent-propagative (Ng and Falk, 2006). *Orthotospoviruses* are transmitted by their thrips vectors in a persistent-propagative manner (Whitfield et al., 2005). In this transmission mode, viruses enter the alimentary canals of their vectors; infect several insect tissues, where they replicate; and then arrive at the PSGs as their final destination (Wei and Li, 2016). Previous studies of *Orthotospoviruses* have demonstrated that TSWV is transmitted in a persistent-propagative manner by *F. occidentalis* (Wijkamp et al., 1996; Margaria et al., 2014), *T. palmi* is a vector species of capsicum chlorosis virus (Chiaki et al., 2020), and WSMoV can be transmitted in a semi-persistent manner by *T. palmi* nymphs but is primarily transmitted in a persistent-propagative manner by adult *T. palmi* after the viruses have been acquired at the first-instar nymph stage (Mou et al., 2021). In this study, we tested the transmission efficiency of different developmental stages of *F. occidentalis* and determined that, like to other *Orthotospoviruses*, TZSV is transmitted in a persistent-propagative manner. This was confirmed by electron micrography of the viroplasm in the PSGs (Figure 2C). However, whether TZSV can be transmitted in a semi-persistent manner remains to be further studied.

To conclusion, our results demonstrated three routes for TZSV transmission in *F. occidentalis* and confirm that, like other *Orthotospoviruses*, TZSV is transmitted in a persistent-propagative manner. These results provide a better understanding of the transmission mode of *Orthotospoviruses*, provide a fundamental basis for further research into the interaction between TZSV and its thrips vector and may aid attempts to control the related diseases.

## Data Availability Statement

The raw data supporting the conclusions of this article will be made available by the authors, without undue reservation.

## Author Contributions

YoC and HW contributed to conception and design of the study. YL, LW, HL, TL, SL, and XZ conducted the experiments. YL, YiC, and HT performed the statistical analysis. YoC and YL wrote the manuscript. All authors contributed to the article and approved the submitted version.

## Funding

This research was supported by grants from the National Natural Science Foundation of China (no. 31871936), the Basic R & D Special Fund Business of Fujian Province (no. 2019R1024-2), the Basic Research Projects of Fujian Academy of Agricultural Sciences (nos. YC2019003, CXTD2021004-3, XTCXGC2021011, CXPT202105, and XTCXGC2021017), and the Yunnan Youth Talent Support of Ten Thousand Talent Program (no. YNWR-QNBJ-2019-156).

## Conflict of Interest

The authors declare that the research was conducted in the absence of any commercial or financial relationships that could be construed as a potential conflict of interest.

## Publisher’s Note

All claims expressed in this article are solely those of the authors and do not necessarily represent those of their affiliated organizations, or those of the publisher, the editors and the reviewers. Any product that may be evaluated in this article, or claim that may be made by its manufacturer, is not guaranteed or endorsed by the publisher.
